# The Complications of Operations for Glands in Neck

**Published:** 1910-04-30

**Authors:** 


### SURGERY.

### THE COMPLICATIONS OF OPERATIONS FOR GLANDS IN NECK.

The removal of enlarged glands in the neck is such a common proceeding and is sometimes so easy that one is apt to overlook the fact that complications of a serious and even alarming nature are occasionally met with. In the first place it is a good rule to exhaust all possible methods of diagnosis before proceeding to operation. To overlook a small primary carcinoma and to remove secondary glands under the impression that they are tuberculous, is not very readily excused. It is useful to remember that primary carcinomata have occurred in such out-of-the-way positions as the anterior and posterior nares and the external auditory meatus, and have only been detected on the post-mortem table. Lymphadenoma is a condition which is sometimes mistaken for tuberculosis and treated accordingly; needless to say, the results bring the surgeon no credit, for speedy recurrence both locally and on the opposite side of the neck, in the groin, or in the axilla is the rule.

The removal of tuberculous glands is best taken as a type, for in the excision of malignant glands anatomical landmarks give place in relative importance to adequate removal of the growth, and the common carotid, external jugular and spinal accessory nerve may all have to undergo ablation in an attempt at a radical operation. In the case of tuberculous glands, the resulting scar is often regarded by the patient as the most important feature of the operation and will be the subject of much anxious inquiry. In order to obtain satisfactory results in this direction three points must be borne in mind. First, an operation must be carried out before the glands have involved the skin—this, of course, is a matter which usually depends upon the patient rather than the surgeon. Secondly, the incision, or incisions, must be planned with care and consideration. They should follow, so far as is convenient, the natural folds of the skin of the neck, and should be so arranged that there is no subsequent tension upon the scar. Lateral tension upon scars makes them stretch, vertical tension probably tends to make them thicken. Lastly, care in suturing is all important. The choice of suture material will depend upon the custom of the surgeon and probably does not matter providing it is clean. But the manner of its introduction does matter. The neck, above all other situations, is one where the sub-cuticular stitch may be used with advantage. Further, it has recently become recognised that the superficial fascia requires consideration; if sutured carefully much of the risk of a stretched, unsightly scar is obviated.

At this date it would seem unnecessary to refer to the importance of rigid asepsis; but when operating on the neck it is very important to remember the proximity of the hair, of the patient's mouth, and of the anæsthetist's hands, and it is necessary to take particular care that neither the hands of the surgeon nor of his assistant (more than one assistant introduces too many elements of risk) touch any of these things. If suppuration does occur, all hope of a satisfactory scar is gone and a long convalescence is inevitable. The large wound, the prolonged manipulation, and the presence of large thin-walled veins render suppuration peculiarly dangerous. Death from toxæmia has resulted within forty-eight hours of one of these operations, owing to general infection by a virulent staphylococcus—so rapid is absorption in this situation.

Severe hæmorrhage, particularly in the case of children, sometimes gives rise to grave anxiety. It usually results from tearing off a small vein at its junction with the internal jugular: a hole in that vessel is produced from which blood escapes in alarming quantities. If the accident happens high up in the neck or low down near the junction with the subclavian, much difficulty may be experienced in applying a lateral ligature or in suturing the rent. It may sometimes be necessary to tie off the main vein; or, if that cannot be done, the wound must be carefully and closely packed with dry ribbon gauze. This should be made with a selvage, to avoid the danger of leaving small portions of cotton thread in the depths of the wound when the dressing is subsequently removed. The extraction of the gauze should generally be attempted after an interval of forty-eight hours, and should be carried out under anæsthesia in case of renewed bleeding and the necessity for further packing. The gauze comes away very easily if a little 10 vol. peroxide of hydrogen be first allowed to percolate through its meshes.

The inevitable division of the small nerves which are met with during the course of the operation does not ordinarily give rise to any trouble, but adult patients occasionally complain of tingling and numbness round the scar subsequently. This usually passes away within a few weeks and requires no active treatment. A complication that has sometimes followed operations on the posterior triangles of the neck, entailing division of the occipital nerves, is the formation of a trophic ulcer, or rather bedsore, on the skin of the occiput. This may be prevented by taking care that there is no continued pressure on any one area of skin owing to the head being kept in one position. The occiput should be carefully examined from time to time, and preferably should rest on an air or water-pillow.

It sometimes happens that the sterno-mastoid muscle has to be divided owing either to the fact that the glands are difficult to remove or that the muscle itself is actually involved in a tuberculous process. It should always be carefully sutured and some form of retentive appliance may be worn to avoid any undue tension on the injured side. A complication which ought not to occur is division of the spinal accessory nerve. A safe plan is to expose this nerve at an early stage, and to keep a watchful eye upon it during the subsequent steps of the operation. Should it be divided, immediate suture must be carried out, for the disability resulting from paralysis of the trapezius is very considerable. It is unfortunate that in many of the cases where it is accidentally divided a considerable section of the nerve is cut away; but suture may even then in some instances be accomplished by splitting the distal portion of the nerve trunk and so lengthening it. Should the accident not be discovered till the wound is healed, it is very questionable whether anything can be done; but if the injury is manifest within a few days of the operation, then it is certainly justifiable to open the wound and attempt to find the divided ends.

